# Production of iron enriched *Saccharomyces boulardii*: impact of process variables

**DOI:** 10.1038/s41598-024-55433-7

**Published:** 2024-02-28

**Authors:** Kiyana Tafazzoli, Mehrdad Ghavami, Kianoush Khosravi-Darani

**Affiliations:** 1 Department of Food Science and Technology, Science and Research Branch, Islamic Azad University, Tehran, Iran; 2 Department of Food Technology Research, Faculty of Nutrition Sciences and Food Technology/National Nutrition and Food Technology Research Institute, Shahid Beheshti University of Medical Sciences, Tehran, Iran

**Keywords:** Box-Behnken, Bioaccumulation, Design of experiments, Enriched yeast, FeY, Probiotic yeast, Process variable, *Saccharomyces boulardii*, Biotechnology, Microbiology

## Abstract

About half of the 1.62 billion cases of anemia are because of poor diet and iron deficiency. Currently, the use of iron-enriched yeasts can be used as the most effective and possible way to prevent and treat anemia due to the ability of biotransformation of mineral compounds into the organic form. In this research, for the first time, *Saccharomyces (S.) boulardii* was used for iron enrichment with the aim that the probiotic properties of yeast provide a potential iron supplement besides improving the bioavailability of iron. Also, due to its higher resistance than other Saccharomyces strains against stresses, it can protect iron against processing temperatures and stomach acidic-enzymatic conditions. So, the effect of three important variables, including concentration of iron, molasses and KH_2_PO_4_ on the growth and biotransformation of yeast was investigated by the Box-Behnken design (BBD). The best conditions occurred in 3 g/l KH_2_PO_4_, 20 g/l molasses and 12 mg/l FeSO_4_ with the highest biotransformation 27 mg Fe/g dry cell weight (DCW) and 6 g/l biomass weight. Such yeast can improve fermented products, provide potential supplement, and restore the lost iron of bread, which is a useful iron source, even for vegetarians-vegans and play an important role in manage with anemia. It is recommended that in future researches, attention should be paid to increasing the iron enrichment of yeast through permeabilizing the membrane and overcoming the structural barrier of the cell wall.

## Introduction

**Figure 1:**
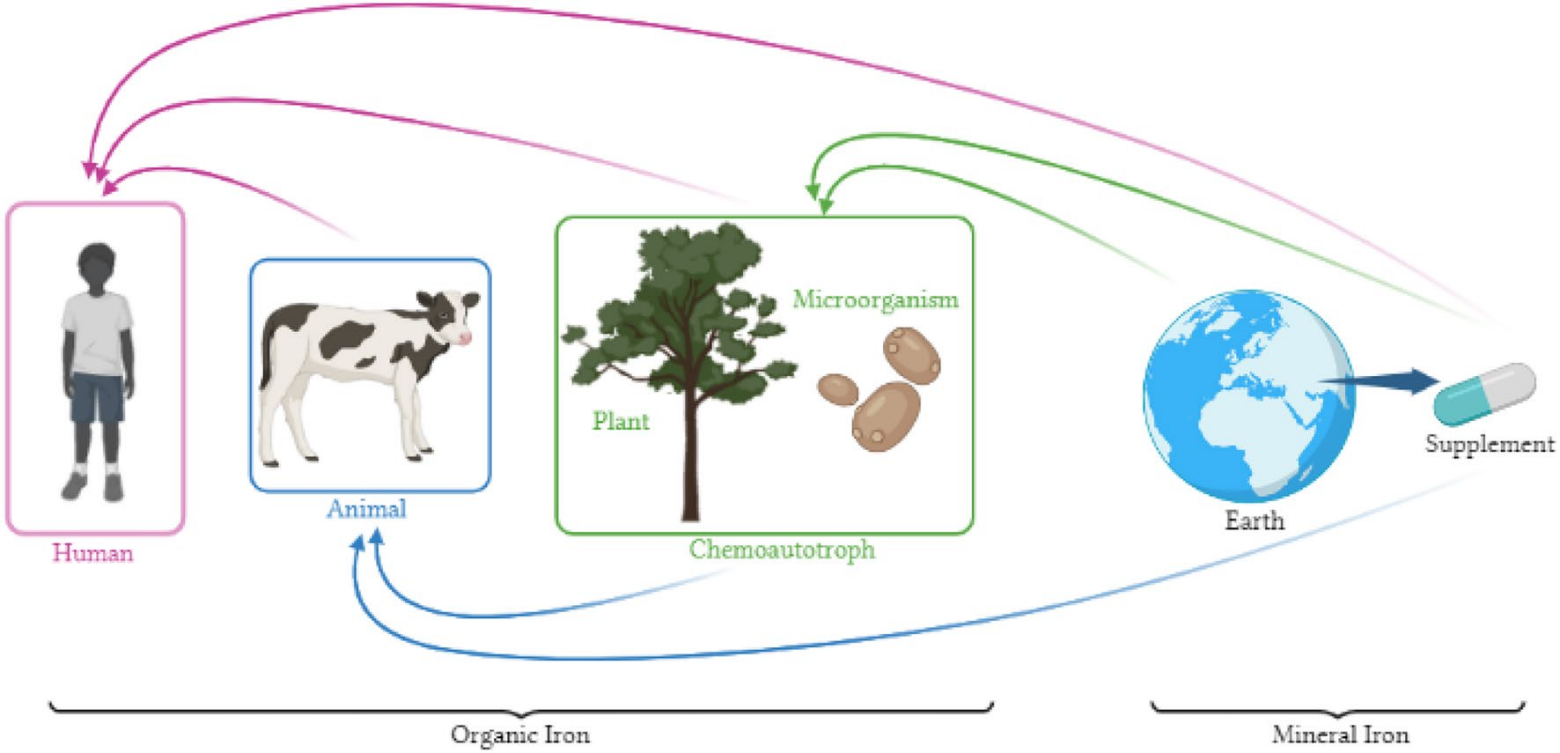
Chain of iron sources. Iron sources are more bioavailable to humans in order from left to right.

Fe in required in various biological and metabolic processes, including DNA synthesis, electron transport, and oxygen transport in eukaryotic cells. So, its deficiency has many health consequences, so it must be supplied by diet^1,2^. Mineral salts are not available for cellular metabolism, so the biological systems of living organisms convert them into an organic and absorbable form. As shown in the food chain in Fig. 1, the chemoautotroph, in the first level of the food chain use inorganic compounds as an energy source and will convert them to organic form during chemical reactions^3^. Animals, will convert Fe into heme. Human diet may be supplied by both of forms. Since iron exists mainly in erythrocytes as the heme compound hemoglobin^2,4^, the iron in animal meat is more absorbable in compare to mineral form. The amount of organic iron in diet cannot meet the all need e.g. in children and pregnant women. So, at risk individuals should consume supplements and fortified foods^5^.

**Figure 2:**
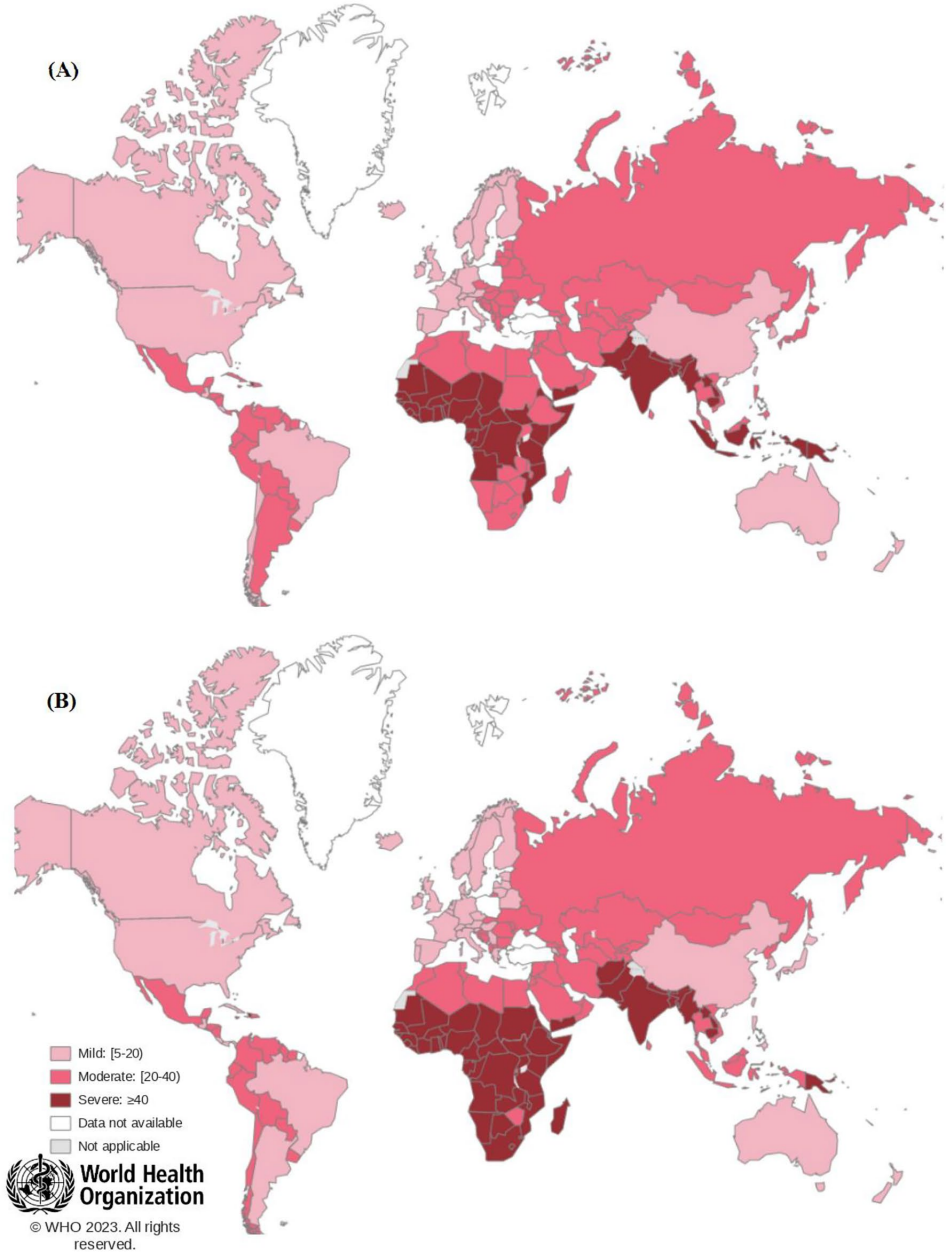
Global prevalence percentage of anemia in (**A**) pregnant women aged 15–49 and (**B**) children aged 6–59 months, by country according to the latest WHO data, 2023.

Based on the latest World Health Organization data in spring 2023, the anemia rate in developed countries has been controlled (Fig. 2)^6,7^. However, the mineral iron, unlike its organic form, has less solubility, so it is not easily absorbed from the digestive tract and has less bioavailability, more toxicity and gastrointestinal side effects^8^. Minerals, when fed in excess, can readily generate free radicals, while organic form has less potential to be toxic^9,10^.

In the meantime, biotransformation is a good approach to overcome problems of mineral iron. Human food chain may be enriched with inorganic iron. Microbial biotransformation is more controllable and possible in microorganisms than in plants and animals^11^.

Yeasts are known as the best option because they have the potential to act as a small and natural biotransformation factory. They absorb the mineral elements enriched in the growth medium under controlled and specific conditions and convert them into organic form by combining with macromolecules of cells^12,13^. Therefore, Iron-fortified yeasts currently being evaluated as a promising source of iron to prevent and reduce iron deficiency in humans and animals^14^. Animal studies have shown that organic iron produced in yeast has a greater potential to absorb and improve anemia than the inorganic form^14,15^. The mechanism of iron accumulation in yeast is vague and needs more research, but studies show when iron is abundant in the extracellular environment, the cell accumulates them in cytosol, mitochondria, nuclei and endoplasmic reticula^16,17^. So, cell is protected from the toxic effect of Fe, and acts as a resource of iron^16–18^. A tiny fraction of iron accumulates as a solution in the cytosol and vacuoles, while a considerable portion (88–68%) is undissolved and chelated to cell constituents^19^. Studies have shown that vacuoles^20^ and cell walls^17^ are the main iron storage compartment. Iron may be complexed with polyphosphate and organic acids in the vacuole^16^.

Organisms with the ability to biotransformation and health-promoting properties could be a good carrier for food micronutrient enrichment. Saccharomyces, has an important role in the production of fermented foods and beverages from ancient due to its high nutritional value, bioavailability, safety, and non-toxicity. So, they have been given much attention in the enrichment and production of organic micronutrients^14,21,22^, as a good source of Fe, especially for vegans and vegetarians. Also, it has great potential in the bread industry. Nutrients, including iron, are lost in the flour production process, so the bread industry has always tried to restore the lost iron^23^. Meanwhile, *S. boulardii* has received much attention because of beneficial probiotic properties and high resistance to stresses during fermentation^24^, processing^25^ and digestion^26^. US Iron enrichment in yeast is limited due to the structure of the cell wall and mannan filaments^27^ and perhaps the characteristics of the iron element, and researches are going to increase it. In this regard, in addition to paying attention to the growth conditions and the genetic pattern^28^ of yeast, efforts are being made to increase the permeability of the cell wall by using methods such as creating protoplasts^13^ and using electric fields^29,30^ and siderophores^12^.

Martínez-Garay et al., studied a large number of *S. cerevisiae* with genetic diversity exposed to different concentrations of iron. They uncovered that the amount of iron accumulation is also highly dependent on their genetic pattern. In such a way that when the iron concentration in the medium increases, iron-sensitive strains in terms of molecular mechanism, accumulate iron more and faster than iron-resistant isolates^28^. Kyyaly el al.^14^ demonstrated that yeast with the 15 mg/g iron accumulation compared to other concentrations tested and inorganic form, achieved the best baking properties (leavening ability). Also Gaensly et al.^31^, concluded that the iron-enriched yeasts maintain its fermentation power. So could be employed as an iron source for supplementing bakery products. But Novsad et al.^29^ they found that the fermentation activity of yeast is reduced when enriched with a large amount of iron, which happened here under the influence of a pulsed electric field, so it can only be used as a medicinal supplement.

BBD is a multivariate graphical tool in statistics and a spherical, powerful 3-level fractional factorial that can determine a complex response function with the small group of parameters. Also, is used to determine the optimal levels of the effective variables and the maximum response and describes the effect of the variables on each other and the response^32^^,^^33^. In this research, for the first time, the *S. boulardii* as an economic, health-giving probiotic was enriched with iron and its biotransformation capacity have been investigated. Also, BBD process was used as one of the powerful common statistical tools of response surface method, to study the combined effect among three important variables (concentration of molasses, iron and KH_2_PO_4_) and finding their optimal levels on iron-biotransformation by yeast.

## Material and method

### Chemicals and media

*S. boulardii* (ATCC 74068) was provided by the (mycology research center, faculty of veterinary medicine, university of Tehran). Molasses was purchased from Bidestan Company (Iran) and analyzed for macronutrient. All the chemicals (ferrous sulfate, monopotassium phosphate, magnesium chloride, peptone, dextrose, agar, hydrochloric acid, nitric acid) were from Merck (German Company).

### Yeast preculture and inoculum preparation

**Figure 3:**
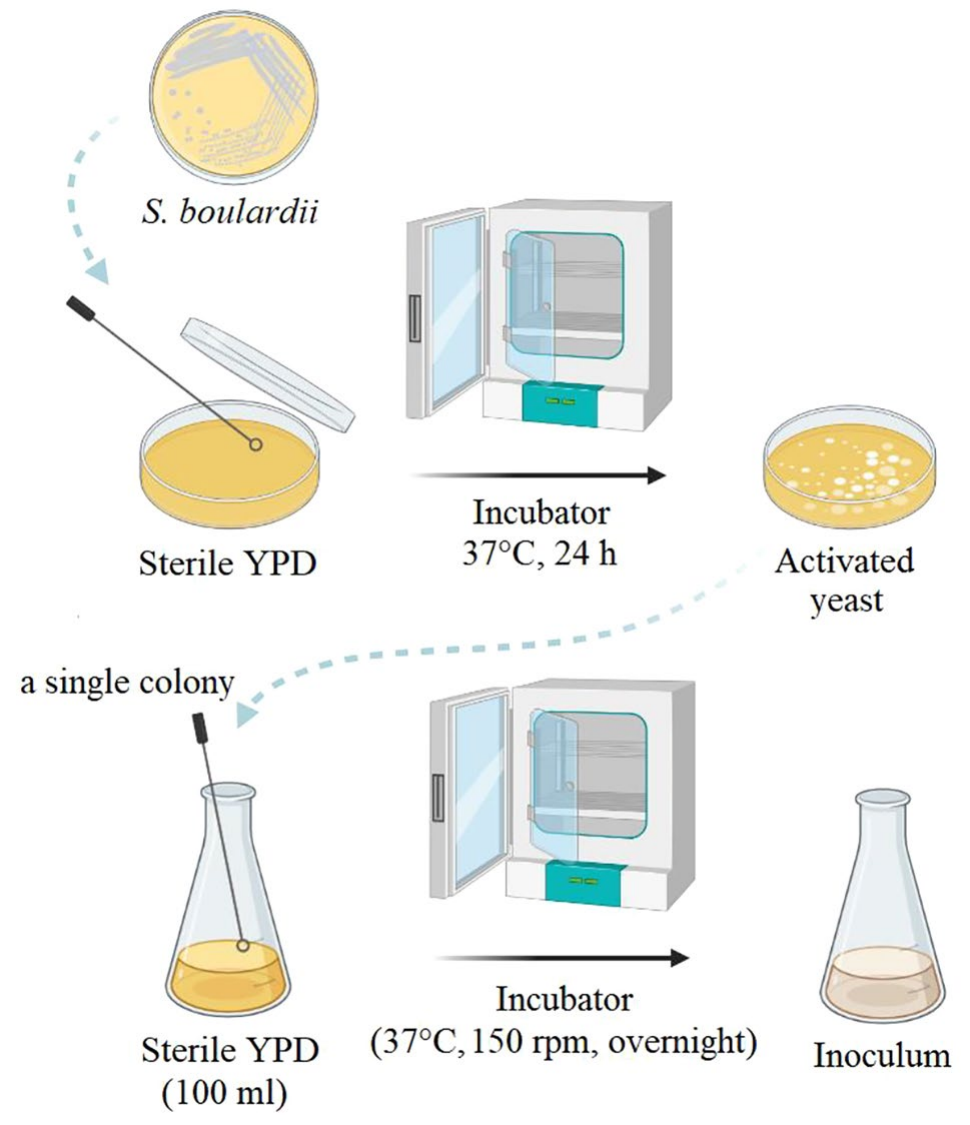
The steps of *S. boulardii* seed liquid preparation.

Yeast was pre-cultured based on Malairuang et al.^34^, with modifications. *S. boulardii* incubated (37 °C, 24 h) in yeast peptone dextrose (YPD) agar medium containing (g/l; peptone: 10, dextrose: 40, agar: 15). To prepare seed culture, a single colony inoculated in 100 ml YPD and incubated (37 °C, 150 rpm) for overnight to near the beginning of the stationary phase, which has the number of living cells (Fig. 3).

### Yeast calibration curve of OD, DCW and cell count of yeast

**Figure 4:**
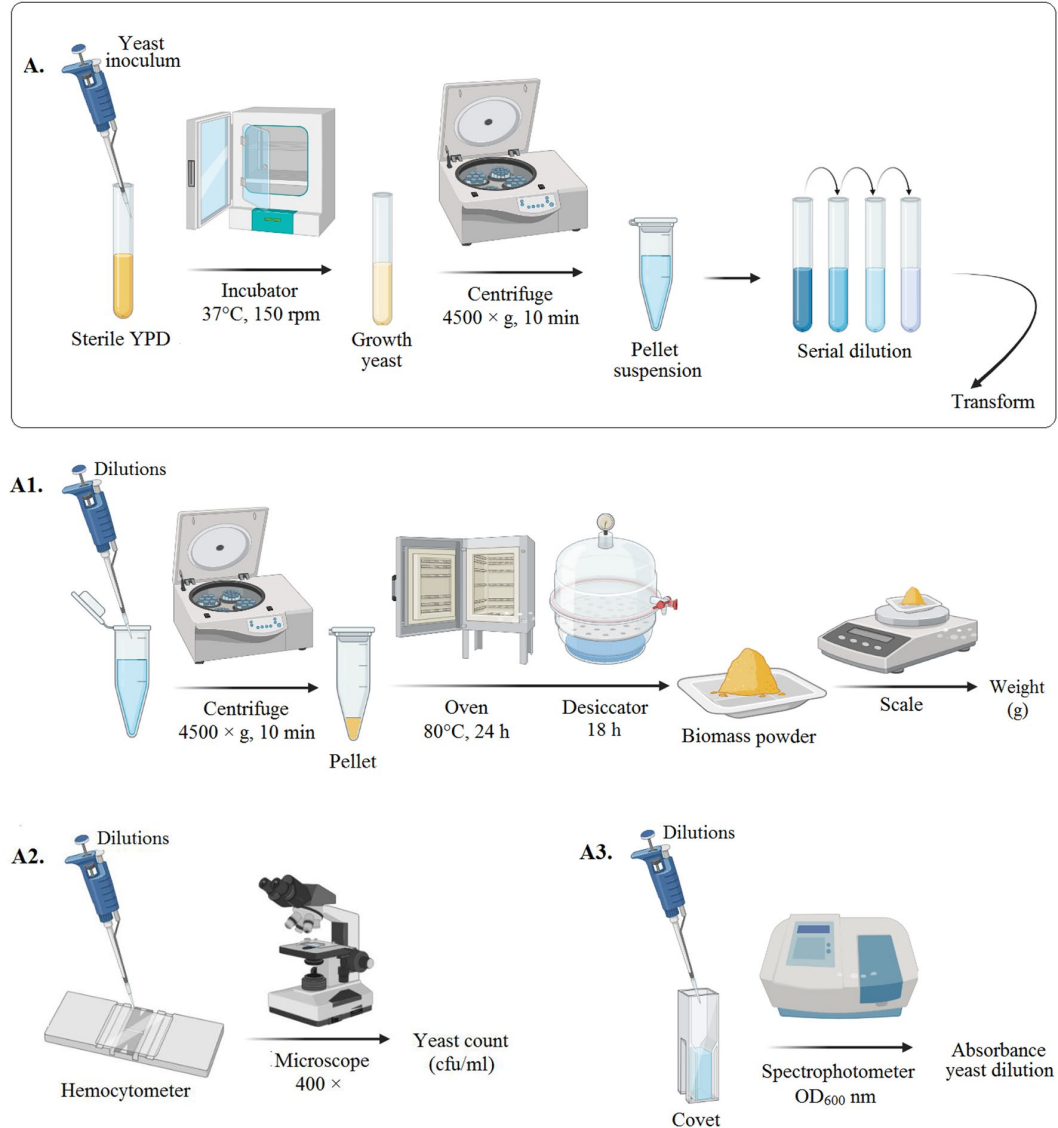
Preparation of serial dilution from the grown *S. boulardii* suspension (**A**) and biomass drying (**A**_**1**_), cell counting (**A**_**2**_) and absorbance reading (**A**_**3**_) of each dilution.

OD and DCW measured based on Malairuang et al.^34^, with some modifications on centrifuge speed. The grown yeast was centrifuged (1500 × g, 10 min). After re-centrifuge and washing the pellet, a serial dilution was prepared from the cell suspension (Fig. 4A). Their absorbance was been measured at OD_600_ nm by a spectrophotometer (PerkinElmer, USA) (Fig. 4A3). Then predefined volume of each dilution was centrifuged and pellet was dried at 80 °C for 24 h to obtain dried cell weight (Fig. 4A1). Also, total count (CFU/ml) was measured by a Thoma hemocytometer (Neubaur chamber) at × 400 on a microscope (CETI; Medline Scientific Limited, Oxfordshire UK) (Fig. 4A2)^35^.

### Yeast growth curve

**Figure 5:**
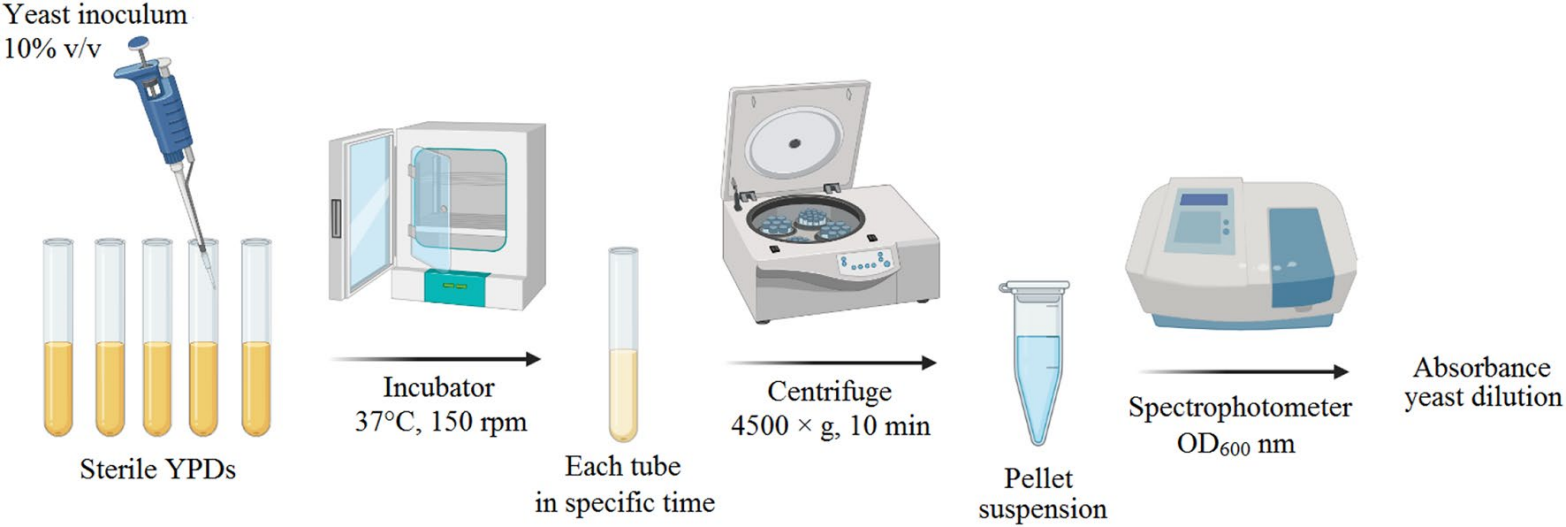
Preparation steps of *S. boulardii* suspension to draw the growth curve.

Cell growth curve was prepared according to Olivares-Marin et al.^36^, with modifications. Tubes containing 9 ml of sterile culture yeast peptone dextrose was prepared, and added 10% v/v seed liquid, then incubate the tubes 37 °C and 150 rpm. At specific intervals, 5 ml of each tube was centrifuged at 1500 g for 5 min. The pellet was washed again and volume reached to 5 ml by adding distilled water. Optical density (OD) was measured at λ 600 nm using a spectrophotometer (Fig. 5).

### Statistical optimization of the significant variables affecting the responses by *S. boulardii* using the Box-Behnken design

**Figure 6:**
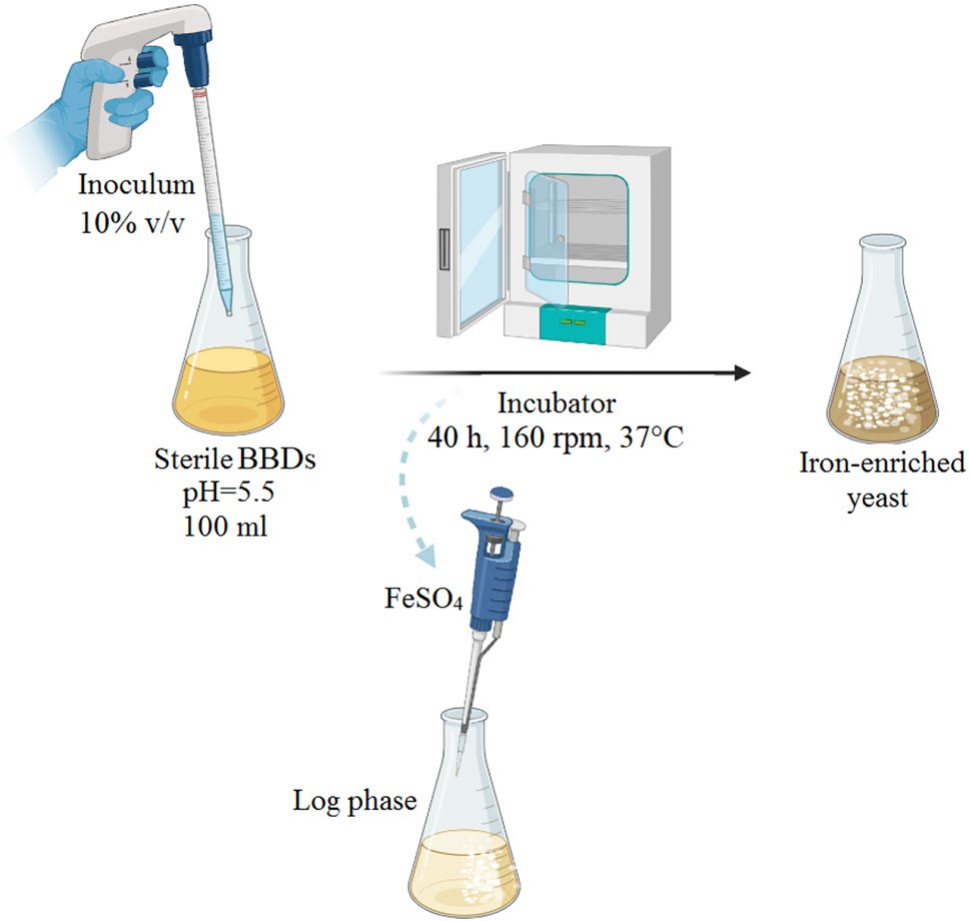
Schematic summary of yeast enrichment at the BBD stage.

In this method, FeSO_4_ and KH_2_PO_4_ as two significant parameters in *S. boulardii* iron biotransformation^37^ (KT) along with molasses as an industrial carbon source, were selected. These independent variables were coded in three levels of low (− 1), medium (0) and high (+ 1), and in 17 runs with five repetitions at the central point to achieve the maximum iron biotransformation, were optimized. According to the Table 1, the medium included molasses, KH_2_PO_4_, MgCl_2_ and peptone, was adjusted at pH 5.5 by HCl and autoclaved at 121 °C for 15 min. Seed liquid volume included 13 × 10^5^ CFU/ml *S. boulardii* was inoculated into the sterile medium and placed in a shaker incubator. FeSO_4_ was added in the beginning of the logarithmic phase. Procedures of experiments have been illustrated in the Fig. 6. The connection between the independent factors and the result to predict the best response is based on the second-order polynomial Eq. (1):

1 $${\text{Y }} = \, \beta_{0} + \mathop \sum \limits_{i = 1}^{k} \beta_{i} X_{i} + \mathop \sum \limits_{i = 1}^{k} \beta_{ii} X_{i}^{2} + \mathop \sum \limits_{i = 1}^{k} \mathop \sum \limits_{j > 1}^{k} \beta_{ij} X_{i} X_{j}$$

where Y is the predicted response; ß_0_, β_i_, β_ii_ and β_ij_ represent the regression constant, linear, quadratic and interaction coefficient, respectively; and X is the independent variable.

**Table 1:** Illustrates the response information, characteristics of each variable and fixed factors for biotransformation by *S. boulardii* using BBD.

	Symbols	Name	Unit	Levels	
				Code −1	Code +1
Variables	A	FeSO_4_	mg/ml	4	12
	B	Molasses (brix 70%)	g/l	15	25
	C	KH_2_PO_4_	g/l	3	6
Fixed factors		MgCl_2_∙6H_2_O	g/l	0.1	
		Peptone	g/l	20	
		Fermentation volume	ml	100	
		Iron adding time	h	Log phase	
		Temperature	°C	37	
		pH	–	5.5	
		Seed liquid volume	(%v/v)	10	
		Agitation speed	(rpm)	160	
		Fermentation time	(h)	40	
		Type of yeast	–	*S. boulardii*	
Responses	R_1_	Biomass Weight	g/l	–	
	R_2_	Organic Iron	mg/l	–

### Dry weight of biomass enriched with iron

**Figure 7:**
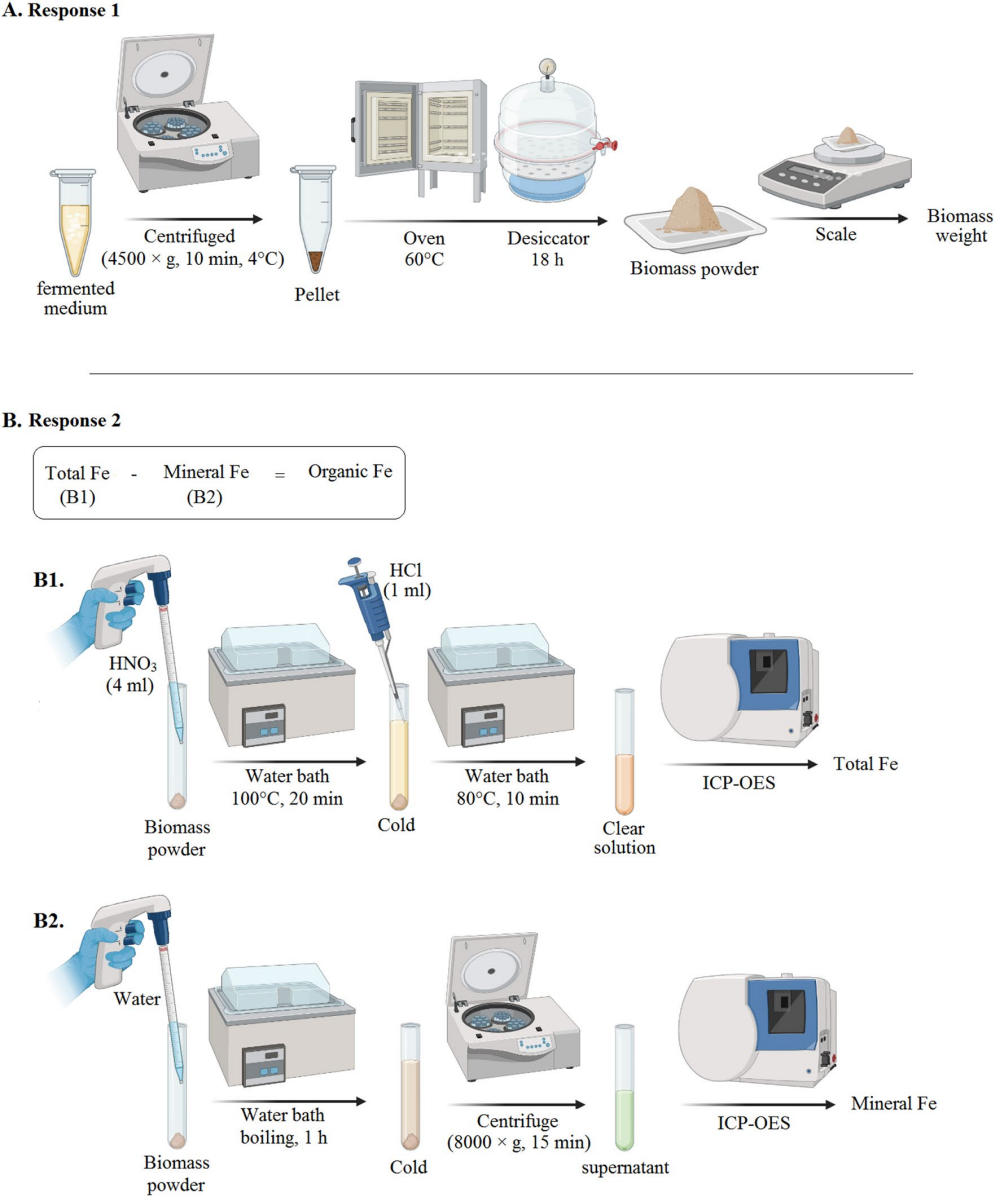
Graphic summary of preparation steps of the response 1; biomass powder and response 2; enriched with iron (**A**) iron biotransformation rate (**B**).

The culture medium and its free mineral iron was removed based on Gaensly et al.^31^, with some modifications on centrifuge conditions. For this aim 10 ml of the fermented medium was centrifuged (1500 × g, 10 min, 4 °C). To ensure the removal of inorganic iron attached to the outer cell’s wall, it was washed three times with deionized water under the above centrifuge conditions. Pellets were dried at 60 °C until they reached a constant weight. Subsequently, they were kept in a desiccator for 18 h to reach equilibrium. The biomass weight in g/l was reported as response (Fig. 7A).

### Determination of organic iron content

Fe determination was performed according to Esmaeili et al.^38^, with some modifications. To measure total iron in yeast, 20 mg of biomass powder was digested in 4 ml of Nitric acid 65–68% v/v at 100 °C for 20 min. In order to complete the digestion process, 1 ml HCl 37% was added to the above cooled solution and incubate for 10 min at 80 °C until a clear solution was obtained (Fig. 7B1). To determine inorganic Fe, 100 mg of biomass powder resuspended in deionized water and boil in a bain-marie for 1 h. After cooling, the solution is centrifuged at (8000 × g, 15 min) supernatant containing mineral iron (Fig. 7B2). Prepared solutions passed through a 0.45u filter and stored in the freezer until the Inductively Coupled Plasma Optical Emission Spectroscopy (ICP-OES) (VISTA-PRO, USA) testing. Finally, by subtracting the amount of inorganic from the total iron, organic content is obtained.

### Statistical analysis

Excel 2016 was used for calculations and drawing graphs. The analysis of variance (ANOVA) and multiple regression of the design of experiment were constructed and analyzed using Design Expert software version 11 (Stat-Ease Inc., USA). Each experiment was performed in duplicate and an average calculated as a response. graphical abstracts were designed by BioRender.

## Result and discussion

### Examination of OD, DCW and cell count of growing yeast over time

Data of yeast calibration curve based on OD, dry cell weight and cell count was summarized in Table 2. also according growth curve (chart 1) logaritmic phase started after 11 h inoculation, and the maximum growth was achieved after 30 h, as also described elsewhere^37^.

**Table 2:** Data of yeast calibration (optical density, cell dry weight, total cell counts).

No.	Absorbance (OD_600 nm_)	Biomass weight (mg)	Cell Number (CFU/ml)
1	0.9	0.7 g	37 × 10^5^
2	0.74	0.6 g	24 × 10^5^
3	0.54	0.3 g	13 × 10^5^
4	0.32	0.3 g	5.1 × 10^5^
5	0.12	0.2 g	0.2 × 10^5^

### Optimization prosses of significant variables influencing on responses using Box-Behnken design

Seventeen runs applied to statistically optimization of the levels of the three variables selected (Table 3). The variation of biotransformation from 122.7 to 161.94 mg/l and biomass weigh from 4.5 to 7.3 g/l shows the acceptable influence of independent variables on the result.

**Table 3:** BBD for evaluation of independent variables on Fe biotransformation and biomass weight.

Std	Run no.	Independent variables			Dependent variables					
		FeSO_4_ (A)	Molasses (B)	KH_2_PO_4_ (C)	Biomass weight (R_1_)			Organic iron contant (R_2_)		
					Actual value	Predicted value	Residuals	Actual value	Predicted value	Residuals
16	1	0	0	0	6.3	6.18	0.12	151.46	151.88	− 0.42
6	2	+ 1	0	− 1	5.9	6.25	− 0.35	161.94	156.49	5.45
3	3	− 1	+ 1	0	5.5	5.55	− 0.05	122.7	121.16	1.54
13	4	0	0	0	5.9	6.18	− 0.28	153.92	151.88	2.04
4	5	+ 1	+ 1	0	7.3	6.95	0.35	152.9	153.35	− 0.44
17	6	0	0	0	6	6.18	− 0.18	155.44	151.88	3.56
11	7	0	− 1	+ 1	6	6.00	0	153.93	148.93	5.00
5	8	− 1	0	− 1	4.6	4.55	0.05	128	124.54	3.46
1	9	− 1	− 1	0	4.5	4.85	− 0.35	129.87	129.42	0.44
14	10	0	0	0	6.1	6.18	− 0.08	150.9	151.88	− 0.98
9	11	0	− 1	− 1	6	5.70	0.30	144.73	148.63	− 3.90
15	12	0	0	0	6.6	6.18	0.42	147.68	151.88	− 4.20
7	13	− 1	0	+ 1	5.7	5.35	0.35	130.59	136.04	− 5.45
8	14	+ 1	0	+ 1	6.4	6.45	− 0.05	149.94	153.40	− 3.46
12	15	0	+ 1	+ 1	6.6	6.90	− 0.3	156	152.10	3.90
2	16	+ 1	− 1	0	6.3	6.25	0.05	145	146.54	− 1.54
10	17	0	+ 1	− 1	6.2	6.20	0	139	144.00	− 5.00
Unit		mg/ml	g/l	g/l	g/l			mg/l		
Level − 1		4	15	3						
Level + 1		12	25	6						

#### The estimated effect of variables

To determine the relationship between the independent variables with each other and responses, the multiple-regression analysis of the BBD was performed in the estimated effect (Chart 2, Table 4). A high positive and negative effect means that a variable has a direct and inverse significant effect on the response, respectively. Since phosphorus, potassium, carbon and iron are essential elements for the growth and activity of microorganisms, all variables had a positive linear effect. But the highest effect was related to the linear coefficient of Iron concentration (A) 12.33 and 0.7 with positive effect followed by its quadratic effect (A_2_) 10.03 and 0.41 with negative effect on the biotransformation and biomass weight, respectively. The linear effect of molasses was the second positive factor affecting biomass weight. While other terms coefficients were not important.

**Table 4:** Show the coefficient estimate of variables on the responses using BBD.

Factor	Coefficient estimate	
	Biomass weight (R_1_)	Biotransformation (R_2_)
Intercept	6.70	146.68
A	0.6750	12.33
B	0.3500	− 0.3663
C	0.2250	2.10
AB	0.0000	3.77
AC	− 0.1000	− 3.65
BC	0.1000	1.95
A^2^	− 0.6500	− 7.43
B^2^	− 0.1500	− 1.63
C^2^	− 0.3500	3.37

#### Analysis of variance (ANOVA) of PBD

For choose the most optimal statistical model matched with the operation results, the ANOVA was employed (Table 5). Predefined F-value, p-value, Confidence Level (CL), R^2^, adjusted (adj) R^2^, predicted (pred) R^2^, adequate (adeq) presision, CV% were used to evaluate the model and prove its accuracy and significance. The analysis, according to low probability value (p < 0.05) for both responses demonstrated that the model was significant. Also the lack-of fits were insignificant which confirmed the validity of the model. The highest magnitude of the effect was related to the linear coefficient of Iron concentration (A) with p-value ≤ 0.001 and high F-value for both responses. Followed by its quadratic effect (A^2^) with coefficient level 99.34% and 95% for biotransformation and biomass weight, respectively. Considering that the identified terms in biotransformation were more significant. The linear effect of molasses was the second factor affecting biomass weight with p < 0.05. While other terms were not significant. The predictive power of the model was indicated by a higher R^2^^39^. And indicates that 90% and 86% of the response variation of biotransformation and biomass weight can be designated by the model, respectively. Adeq precision with a ratio > 4 is desirable, which in our research was about 8 for both responses, implying that the model generated an adequate signal to design space^40^. The lower C.V.% indicated good reliability of the experimental values^41^.

**Table 5:** The ANOVA for the experimental results of BBD.

Terms	Biomass weight (R1)						Organic iron content (R2)					
	df	Sum of squares	Mean Square	F-value	p-value	Confidence level%	df	Sum of squares	Mean Square	F-value	p-value	Confidence level%
Model	9	6.38	0.7	5.02	0.0224	97.76%	9	1896.52	210.72	7.21	0.0081	99.19%
A (FeSO_4_)	1	3.92	3.92	27.77	**0.001**	99.9%	1	1215.74	1215.74	41.62	**0.0003**	99.97%
B (Molasses)	1	0.98	0.98	6.94	**0.0337**	96.63%	1	1.07	1.07	0.03	0.8534	14.66%
C (KH_2_PO_4_)	1	0.5	0.5	3.54	0.1018	89.82%	1	35.24	35.24	1.21	0.3084	69.16%
AB	1	0	0	0	1	0.00%	1	56.78	56.78	1.94	0.2059	79.41%
AC	1	0.09	0.09	0.63	0.4508	54.92%	1	53.22	53.22	1.82	0.2191	78.09%
BC	1	0.04	0.04	0.28	0.611	38.9%	1	15.21	15.21	0.52	0.4939	50.61%
A^2^	1	0.72	0.72	5.14	**0.05**	95.00%	1	423.58	423.58	14.50	**0.0066**	99.34%
B^2^	1	0.07	0.07	0.54	0.4849	51.51%	1	75.43	75.43	2.58	0.1521	84.79%
C^2^	1	0.05	0.05	0.39	0.5499	45.01%	1	2.48	2.48	0.08	0.7792	22.08%
Residual	7	0.98	0.14				7	204.45	29.21			
Lack of fit	3	0.68	0.22	2.94	0.162	8.38	3	168.84	56.28	6.32	0.535	5.35%
Pure error	4	0.3	0.07				4	35.61	8.9			
Cor total	16	7.37					16	2100.97				
	Std. dev.		0.3757	R^2^		0.8659	Std. dev.		5.4	R^2^		0.9027
	Mean		5.99	Adj R^2^		0.6936	Mean		145.53	Adj R^2^		0.7776
	C.V.%		6.27	Pred R^2^		− 0.5417	C.V.%		3.71	Pred R^2^		− 0.3123
				Adeq Precision		8.3293				Adeq precision		8.5254

The significant P-value are written in bold.

#### Regression equation

The regression coefficients were calculated (Table 4) and results were fitted to a second-order polynomial function to define the best optimization algorithm. So, simplification of the regression equation after removal of non-significant terms gave the reduced Eq. (2):

$${\text{R}}_{{\text{biomass weight}}} = { 6}.{18 } + \, 0.{\text{7 A }} + \, 0.{\text{35 B }}{-} \, 0.{\text{41 A}}^{{2}}$$

$${\text{R}}_{{\text{organic iron}}} = { 151}.{88 } + { 12}.{\text{33 A }}{-}{ 1}0.0{\text{3 A}}^{{2}}$$

where R is the response and the A is the FeSO_4_ concentration factor.

#### Model adequacy checking

The normal probability plot of residuals and predicted vs observed plot for two studied responses was linear and points gathered around the diagonal line and which confirming the model’s validation. (Chart 3).

#### Examining the interaction effect of variables

Three-dimensional response surface plots were used to visualize the main and mutual effects of the variables on the responses simultaneously, assuming that the third variable remains constant at the central point (chart 4). All three variables of FeSO_4_, KH_2_PO_4_ and molasses had an increasing interaction effect on the responses. Generally, medium stability, appropriate concentration, availability, recovery, preserving the soluble and absorbable state of compounds, their strengthening effect, overcoming the thick wall of oligosaccharides and production of metabolites effective in absorption, can effective on cell growth and biotransformation. Reasons are not exactly clear here, but could certainly be because of the key role of the variables in many biological processes that led to their effective interaction in the metabolic pathways of cell growth and increased permeability or mechanisms related to iron absorption. Possible cases are mentioned:

KH_2_PO_4_ and molasses have multiple positive interaction on yeast growth (chart 4A) which may be due to mechanisms: (1) phosphorus, potassium and carbon play important roles in metabolic pathways and cellular structures, which may reinforce each other's effect. For example, phosphate is a cofactor required by some enzymes^42^ and carbon plays a role in providing ATP for reactions, so their simultaneous use may affect the better regulation of enzyme activity and metabolism, which leads to improved growth. (2) The medium pH decreases rapidly in the early stages of fermentation and in the presence of nutritional sources. The main reasons for the pH fall during fermentation are unclear. But studies have shown that the excretion of acids such as carbon dioxide, organic acids, acidic nucleotides or hydrogen ions are effective. So KH_2_PO_4_ and molasses can also have an indirect positive interaction because of the effect on yeast growth in reducing pH. Also, the addition dibasic potassium salt, although it initially leads to a rise in medium pH, but the yeast rapidly altering the buffering capacity by assimilating bases such as phosphate leads to an increase in acidity^43^. The pH rising can stimulate yeast to consume more of certain nutrients like iron with some mechanisms. For instance, at physiological pH, soluble ferrous iron (Fe^2+^) is quickly oxidized to the insoluble ferric (Fe^3+^) form, but acidic conditions can prevent this process and increase iron solubility and absorption. Also, due to the increased secretion^44,45^ and activity of hydrolase such as invertase in acidic conditions^46^, the extracellular sucrose digestion into usable carbohydrates (glucose and fructose) increases. Among common sugars, fructose has an exceptional chelating ability and readily combines with metal ions such as iron^47,48^. Unlike high-molecular-weight carbohydrate complexes, non-heme iron complexes with simple sugars such as fructose easily penetrate biological membranes^47,48^ and increases bioavailability and absorption significantly compared to iron alone^49^. Which can have a mutual effect on growth along with increasing molasses and providing more polysaccharide content.

About biotransformation (chart 4D), these two variables had a mutual additive effect on the response up to a certain concentration of carbohydrates provided by molasses (range 21 g/l). And then absorption decreased, probably due to the increase in iron absorption inhibitors in molasses, including polyphenols^50–52^, which occurs through mechanisms such as insoluble iron-polyphenol complexes^53^. The results show that in a suitable and constant molasses concentration, iron absorption depends almost on phosphorus concentration, and with it added, the response increased with a shallow slope. Probably because potassium with decreasing acidity has increased the iron bioavailability and the production of fructose, which are effective in iron absorption. Comparing these two responses, we see that the increase of molasses did not have the similar increasing effect. With increasing molasses to the effective level at the growth peak (chart 4A), iron biotransformation decreased, which can be explained by the different enhancing and inhibiting effects of molasses on the two responses.

FeSO_4_ and KH_2_PO_4_ had a positive interaction on biomass weight (chart 4B) that can be caused by: (1) considering the role of iron and constituent elements of KH_2_PO_4_ in many biological processes, their interaction may be effective in cell growth. (2) Phosphate increases the solubility and cellular absorption of iron by indirectly lowering the pH, so Fe required for cell growth is provided on a larger scale. The highest response was observed at 10 mg/ml FeSO_4_ and 4.8 g/l KH_2_PO_4_, then it decreased. It may be because of the negative effect of iron overload as a pro-oxidant agent that increases reactive oxygen species (ROS) and saturates the antioxidant system. The toxicity-mediated ROS causes cellular changes such as damage to the plasma membrane and intracellular organelles, disruption of biochemical processes and cellular homeostasis, leading to ferroptosis^54–56^. Also, increasing KH_2_PO_4_ by causing acid stress can negatively affect the proteins activity and cell growth, accelerate the ferroptosis process and even act as a limiting factor in iron absorption. Of course, the toxicity caused by the accumulation of cell excreta, lack of nutrients is also not ineffective in reducing cell growth.

Chart 4E shows that in the range of 4–6 mg/ml iron, increasing phosphate slight increased iron absorption. This is because KH_2_PO_4_ causes changes in the iron physicochemical properties by indirectly reducing the pH and facilitates its absorption. But the KH_2_PO_4_ addition did not have an obvious effect on an iron element in increasing its absorption. And all KH_2_PO_4_ concentrations led to the highest response in the best range of FeSO_4_ (9–12 mg/ml). It is probably due to the suitable pH of the medium in all KH_2_PO_4_ concentrations, which kept most of the irons in an absorbable state and led to the relative ineffectiveness of changing the potassium concentration in iron absorption. Compared to the biomass weight, although the increase of iron in low potassium conditions was associated with an increase in iron absorption (chart 4E), but the supply of cellular iron in low potassium conditions was not enough for yeast growth (chart 4B). Which is probably caused by the effect of potassium on factors other than iron that stimulated cell growth.

FeSO_4_ and molasses also had a reinforcing effect on biomass weight, which can be due to: (1) Iron can improve the processes in which carbon participates as the main component, such as carbon pathways and cellular respiration, as a catalyst helper and the main component of complexes^57^. (2) Iron may also be used as an oxidant during the ferrous iron respiration, which leads to increased yeast activity and absorbs more carbon to growth. In chart 4C the positive linear effect of iron and molasses and the negative quadratic effect of iron are evident.

It was also seen in the chart 4F that iron absorption reached its maximum in a period of nearly 10.5 mg/ml molasses and 21 g/l FeSO_4_ with an obvious quadratic effect. Which shows the increasing effect of FeSO_4_ and molasses correlation on yeast iron absorption. An iron-rich diet can definitely contribute to iron accumulation, but the graph showed that in the conditions of high iron and low molasses, iron absorption was low. Which is probably due to the significant and strengthening role of simple carbohydrates in the mechanisms of iron transport and absorption. Considering that studies have shown that fructose forms soluble but relatively stable complexes with both ferrous and even insoluble ferric iron^47,48^ and significantly increases their absorption and bioavailability^49^.

The results show that the interaction between molasses and FeSO_4_ compared to other variables in the two answers is almost identical, and the responses at 4.5 g/l KH_2_PO_4_ enhance with increasing FeSO_4_ and molasses (chart 4C, F). The clear curvature of iron (all iron) in the meshes shows that their concentration range is chosen almost appropriately.

#### Multi-response optimization using the desirability function

Both biomass weight and biotransformation responses were considered for multi-response optimization using the overall desirability function. The two-dimensional contour plot of the composite desirability function is the geometric average of the individual function of each response. Which it shows the interaction of two most effective variables (FeSO_4_ and molasses) on the set of responses. While KH_2_PO_4_ was constant at its optimum point (Chart 5B). As shown, responses increased with the adding in molasses and FeSO_4_ and it reaches the maximum in 10.54 mg/l FeSO_4_.

According to the desirability ramp (Chart 5A), the model predicts that the optimal responses are provided in the 10.5 mg/l FeSO_4_ and the highest amount of molasses and KH_2_PO_4_. Also, the validation experiments confirmed the predicted values at a modified optimal condition.

## Conclusion

In this study, BBD use to optimize three variables iron, molasses and KH_2_PO_4_ concentration for maximum biomass weight and biotransformation of *S. boulardii*. The optimum responses were 156 mg/l and 7 g/l for biotransformation and biomass weight, respectively at the highest levels of KH_2_PO_4_ and molasses and 10.54 mg/l FeSO_4_. But the maximum biotransformation (161.94 mg/l) was observed in 12 mg/l FeSO_4_, 20 g/l Molasses and 3 g/l KH_2_PO_4_ with 6 g/l biomass weight, which was considered as the best condition.

This enriched *S. boulardii* can be recommended as a medicinal supplement or incorporated into bread to prevent and treat anemia. According to United States Department of Agriculture in 2018 the standard amount of iron in each slice of bread with average weight equal to 1.8 mg, also 2% w/w yeast is needed for bread production. Produced yeast in this research can provide 1/3 standard iron of bread. Low iron enrichment in yeast can be due to the nature of this element and the steric hindrance of boulardii mannans. So, to increase the biotransformed iron and more industrial applications, it is recommended to investigate the factors affecting the permeability of the membrane and overcome the oligosaccharide barrier of the cell wall along with the investigation of its fermentation properties in future research.

### Supplementary Information

Supplementary Figures.

## Data Availability

The datasets used and/or analysed during the current study available from the corresponding author on reasonable request.
