# Supplementary material for: Production of iron enriched Saccharomyces boulardii: impact of process variables
Source: Sci Rep. 2024 Feb 28;14:4844. doi: 10.1038/s41598-024-55433-7 (PMC10902395; doi:10.1038/s41598-024-55433-7)
Supplement: Supplementary file 1 — Supplementary Figures. [file 41598_2024_55433_MOESM1_ESM.docx]

**Chart 1.** Growth curve of yeast *Saccharomyces cerevisiae* in peptone dextrose agar medium

**Chart 2.** Statistical analysis illustrates the main effect of independent factors on the responses using BBD.

**Chart 3**. (A, B) Plot of the correlation between predicted versus observed values and (C, D). The normal probability plot of the studentized residuals, for biotransformation and biomass weight determined by the first-order polynomial equation.

**Chart 4.** 3D-response surface plots demonstrate the interaction between two variables (X-axis) and the response (Y-axis) simultaneously, KH_2_PO_4_-molasses on R1 (A), R2 (D), KH_2_PO_4_-FeSO_4_ on R1 (B), R2 (F) and molasses-FeSO_4_ on R1 (C), R2 (F), while the third variable is fixed at its center point.

**Chart 5.** Graphic representation of (a) Desirability ramp: indicating the optimal value of variables and responses with an overall desirability score of 0.884. b) The 2D plot of the desirability function for optimal settings: the interaction effects of two variables (X and Y axes) in the constant potassium value at the optimal point on the average responses are presented simultaneously.

**Chart 1.** Growth curve of yeast *Saccharomyces cerevisiae* in peptone dextrose agar medium

**Chart 2.** Statistical analysis illustrates the main effect of independent factors on the responses using BBD.


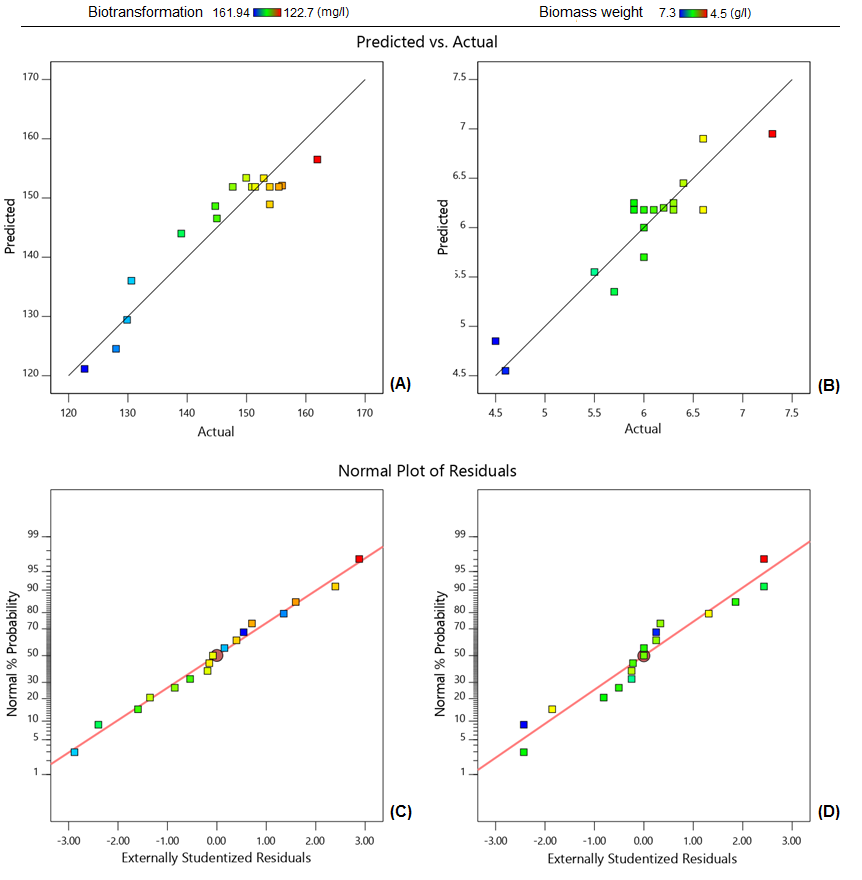
**Chart 3**. (A, B) Plot of the correlation between predicted versus observed values and (C, D). The normal probability plot of the studentized residuals, for biotransformation and biomass weight determined by the first-order polynomial equation.

**
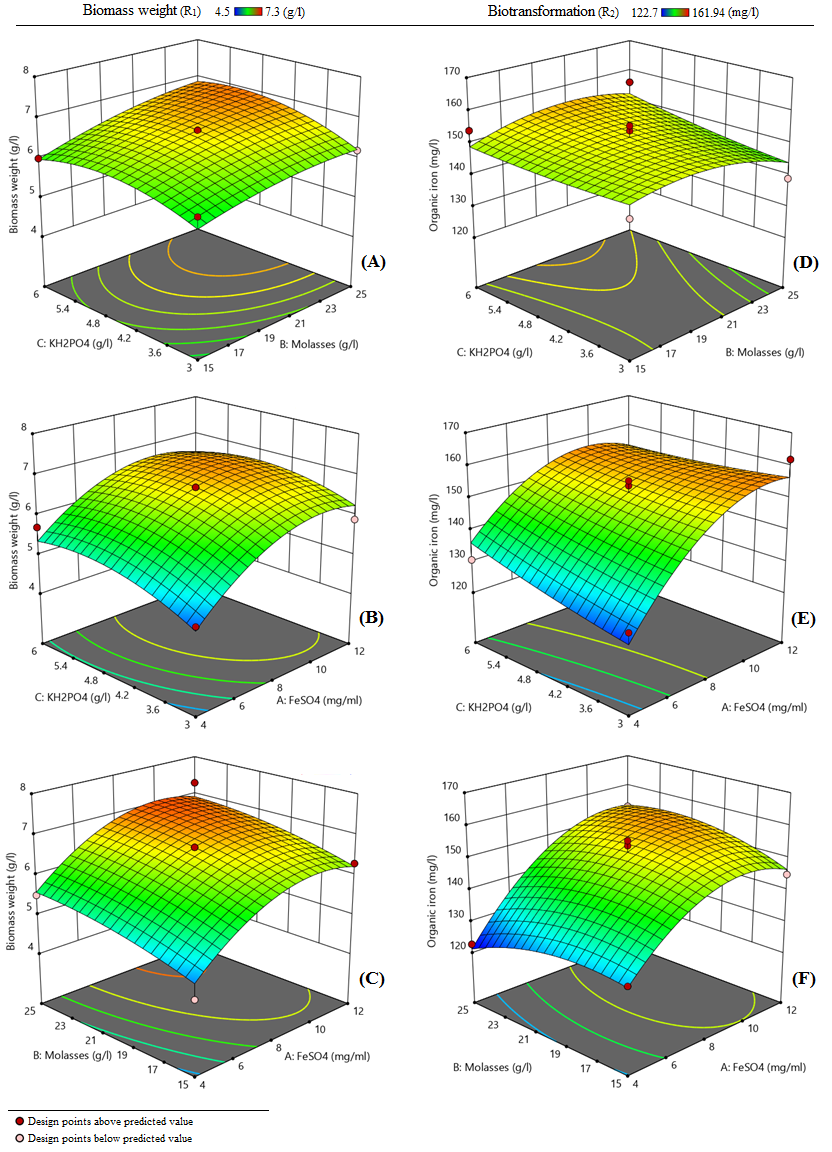
**

**Chart 4.** 3D-response surface plots demonstrate the interaction between two variables (X-axis) and the response (Y-axis) simultaneously, KH_2_PO_4_-molasses on R1 (A), R2 (D), KH_2_PO_4_-FeSO_4_ on R1 (B), R2 (F) and molasses-FeSO_4_ on R1 (C), R2 (F), while the third variable is fixed at its center point.

**
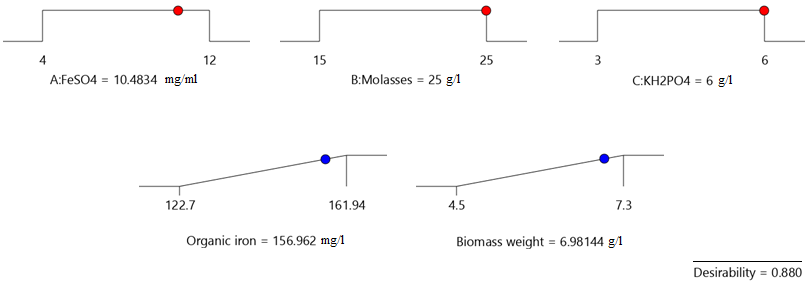
A.**

**B.**


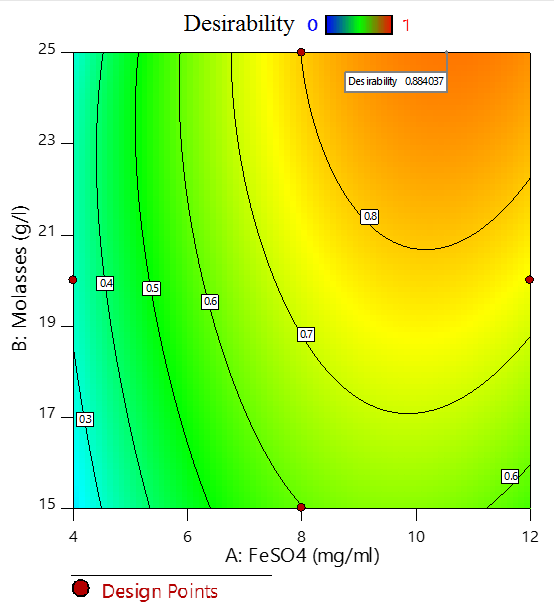


**Chart 5.** Graphic representation of (a) Desirability ramp: indicating the optimal value of variables and responses with an overall desirability score of 0.884. b) The 2D plot of the desirability function for optimal settings: the interaction effects of two variables (X and Y axes) in the constant potassium value at the optimal point on the average responses are presented simultaneously.
